# Regulatory Network of Secondary Metabolism in *Brassica rapa*: Insight into the Glucosinolate Pathway

**DOI:** 10.1371/journal.pone.0107123

**Published:** 2014-09-15

**Authors:** Dunia Pino Del Carpio, Ram Kumar Basnet, Danny Arends, Ke Lin, Ric C. H. De Vos, Dorota Muth, Jan Kodde, Kim Boutilier, Johan Bucher, Xiaowu Wang, Ritsert Jansen, Guusje Bonnema

**Affiliations:** 1 Wageningen UR Plant Breeding, Wageningen University & Research Centre, Wageningen, The Netherlands; 2 BU Bioscience, Plant Research International, Wageningen, The Netherlands; 3 Centre for BioSystems Genomics, Wageningen, The Netherlands; 4 Netherlands Metabolomics Centre, Leiden, The Netherlands; 5 Groningen Bioinformatics Centre, Groningen Biomolecular Sciences and Biotechnology Institute, University of Groningen, Haren, The Netherlands; 6 Institute of Vegetables and Flowers, Chinese Academy of Agricultural Sciences (IVF, CAAS), Beijing, China; Nanjing Agricultural University, China

## Abstract

*Brassica rapa* studies towards metabolic variation have largely been focused on the profiling of the diversity of metabolic compounds in specific crop types or regional varieties, but none aimed to identify genes with regulatory function in metabolite composition. Here we followed a genetical genomics approach to identify regulatory genes for six biosynthetic pathways of health-related phytochemicals, i.e carotenoids, tocopherols, folates, glucosinolates, flavonoids and phenylpropanoids. Leaves from six weeks-old plants of a *Brassica rapa* doubled haploid population, consisting of 92 genotypes, were profiled for their secondary metabolite composition, using both targeted and LC-MS-based untargeted metabolomics approaches. Furthermore, the same population was profiled for transcript variation using a microarray containing EST sequences mainly derived from three *Brassica* species: *B. napus*, *B. rapa* and *B. oleracea*. The biochemical pathway analysis was based on the network analyses of both metabolite QTLs (mQTLs) and transcript QTLs (eQTLs). Co-localization of mQTLs and eQTLs lead to the identification of candidate regulatory genes involved in the biosynthesis of carotenoids, tocopherols and glucosinolates. We subsequently focused on the well-characterized glucosinolate pathway and revealed two hotspots of co-localization of eQTLs with mQTLs in linkage groups A03 and A09. Our results indicate that such a large-scale genetical genomics approach combining transcriptomics and metabolomics data can provide new insights into the genetic regulation of metabolite composition of *Brassica* vegetables.

## Introduction

*Brassica* crops are important food sources (oil, vegetables and condiments), and are considered to have beneficial nutritional properties such as antitumoral activities [1], [2], [3]. The presumed healthy components such as phenylpropanoids, phenolics, flavonoids and glucosinolates have been widely characterized in *Brassica* [4], [5], [6]. For example, *Brassica* leaves have been found to accumulate conjugated forms of both flavonols (quercetin, kaempferol and isorhamnetin) and flavones (apigenin and luteolin) [5]. In recent years, breeding for nutritional quality became an important research topic and in this context metabolomics approaches, using NMR, LC-MS and or GC-MS, have enabled the parallel assessment of the relative levels of a broad range of metabolites [7], [8], [9], [10]. In *Arabidopsis*, a *Brassica rapa* relative, the metabolite variation was found to be abundant and its genetic regulation complex, plausible candidate regulators could be identified after LC-MS mass peaks were assigned to genomic loci [11].

The components of the metabolome can be regarded as the end products of expressed genes and define the biochemical phenotype of a cell or tissue. Several complementary analytical methods can be applied in order to enable profiling, either relatively or quantitatively, of the various chemical classes of metabolites present in an organism [12], [13].

Transcriptomics measures the variation in mRNA transcript abundance and expression profiles can be treated as heritable traits to genetically map expression quantitative trait loci (eQTL); this type of analysis has been denominated as genetical genomics [14].

Thus, the integration of metabolomics with transcriptomic and genomic platforms has frequently been used as a strategy to identify candidate genes involved in the regulation of the levels of specific metabolites in plant systems [11] [15], [16], [17], [18]. The investigation of selected biochemical pathways of pre-defined metabolites showed that the connections between gene expression and metabolite variation are complex [19], [20].

The variation in *B. rapa* morphology is huge (oil, turnip, pak choi, Chinese cabbage and several Asian leafy morphotypes) and the variation in metabolite composition is similarly large [21], [22], [23]. This variation has increased the interest of plant breeders to breed for improved phytonutrient quality in *Brassica*. Several studies in *A. thaliana*, *B. rapa* and *B. napus* aimed to identify QTL for phytonutrients. The most studied secondary metabolites are the glucosinolates and the detected QTL not only confirmed the quantitative nature of this trait, but also allowed the identification of key biosynthetic and regulatory genes (Hasan et al. 2008, Kliebenstein, 2001,Lou et al. 2008, Wentzel et al. 2007, Feng et al. 2012).

The triplicated genome of *B. rapa* has a well described synteny with *Arabidopsis* [24], [25], [26]. As a consequence of the evolutionary gene triplication event, many genes have paralogues. The triplicated nature of the *Brassica* genome represents a challenge to unravel the genetics of metabolic traits using genetical genomics approaches.

In the present study we profiled both metabolite and transcript abundance in the leaves of six weeks-old plants from a Doubled Haploid (DH) population developed from an F1 cross between a yellow sarson (R500) and a pak choi (PC175) plant. We applied both an untargeted metabolomics approach for semi-polar secondary metabolites, including glucosinolates, flavonoids and phenylpropanoids, using liquid chromatography-mass spectrometry (LC-MS), and targeted analytical approaches to profile lipid-soluble isoprenoids (carotenoids and tocopherols) and folates. Additionally, the whole genome transcript level was performed on leaves of six week old plants from all DH lines using a distant pair design with a 60-mer oligo microarray assembled using EST sequences mainly from three species: *B. napus*, *B. rapa* and *B. oleracea* [27]. To prioritize the number of candidate genes, we subsequently focused on six known biochemical pathways of health-related phytonutrients, with the aim to identify regulatory genes. Furthermore, for the construction of a transcriptional regulatory network we used the very well described glucosinolate pathway [28], [29]. These data are an important reference for breeding purposes and a step to gain insight in the genetic factors responsible for the metabolite variation in *B. rapa*.

## Materials and Methods

### Parental materials to develop a doubled haploid (DH) population

A *B. rapa* DH population was developed from a cross between pak choi PC-175 (cv.Nai Bai Cai; accession number VO2B0226), as the male parent, and yellow sarson YS-143 (accession number FIL500). The parental accessions were selected based on their differences in phenotypic characteristics and genetic distance [30], [31]. Actually, this population is a reciprocal cross of the previously developed population DH38 as described in [31].

The DH population was created using the microspore culture protocol described in [31], [32] [33] and [34]. The progeny of the DH plants from three F1 plants were used for the phenotyping and genotyping. The resulting population was named DH68 and consisted of 92 DH lines and for each line the corresponding F1 parent was known.

### Plant growth conditions

The plants of the DH lines were grown in the greenhouse under the following conditions: 16 hrs light and temperature between 18 and 21°C. After one week, germinated seedlings were transplanted and randomly distributed over three different blocks. Five weeks after transplanting, the 3^rd^ and 4^th^ leaves of each replicate were collected and immediately frozen in liquid nitrogen to be grinded into fine powder and stored at −70°C. Each replicate was grinded individually and a pool of equally weighed amount of each of the three replicates was used for metabolic and transcriptomic profiling, as well as for DNA marker profiling to construct a linkage map and perform QTL analysis.

### Construction of a genetic linkage map

Leaf material for DNA extraction was collected from seedlings and then ground using a Retsch 300 shaker (Retsch BV, Ochten, the Netherlands). DNA was isolated based on modified CTAB methods [35]. The AFLP analysis was performed according to Vos *et al*. (1995) [36].The AFLP and marker primer combinations used for the mapping were those combinations used for map construction in [37]. Linkage analysis and map construction were carried out using JoinMap 4.0 (http://www.kyazma.nl) for each population. Linked loci were grouped on the basis of pairwise LOD values between 4 and 7. The Kosambi mapping function was used to convert recombination data to map distances.

### LC-MS metabolic profiling

Leaf samples were analyzed for variation in semi-polar metabolite composition using LC-QTOF-MS, essentially as described in [10]. In short, 0.5 g FW of frozen leaf powder of each DH line and the parents was extracted with 1.5 ml of methanol containing 0.1% formic acid. Samples were sonicated and then filtered (Captiva 0.45 µm PTFE filter plate, Ansys Technologies) into 96-well plates with 700 µl glass inserts (Waters) using a TECAN Genesis Workstation equipped with a 4-channel pipetting robot and a TeVacS 96-wells filtration unit. Samples were injected (5 µl) using an Alliance 2795 HT instrument (Waters), separated on a Phenomenex Luna C18 (2) column (2.0×150 mm, 3 mm particle size) using a 5–35% acetonitrile gradient in water (acidified with 0.1% formic acid) and then detected on-line firstly by a Waters photodiode array detector (wavelength 220–600 nm (Waters) and secondly by a Water-Micromass QTOF Ultima MS with negative electrospray ionization (m/z 80–1500). Leucine enkephalin was used for online mass correction.

Metalign software (www.metalign.nl) was used to automatically extract and align all relevant mass signals (signal to local noise ratio >3) from the raw data files. The total of 6,673 mass peaks was filtered for signals being present in at least 15 samples and having amplitudes of at least 100 ion counts per scan (about 6 times the noise value) in at least one of the samples. Then, mass signals originating from the same metabolites were clustered based on their similar retention times and variation over samples, using MSClust software [13]. This retained 228 so-called centrotypes, or reconstructed metabolites, of which the relative abundance was represented by the total ion counts of the clustered signals.

### Targeted analyses of folates and lipid-soluble isoprenoids

Lipid soluble isoprenoids, including carotenoids, tocopherols and chlorophylls, were extracted using Tris-buffer/methanol/chloroform and analyzed by HPLC-PDA-fluorescence as described before [21]. Folates were extracted in a Na-acetate buffer pH 4.7 containing 20 µM DTT, and total folate levels were determined using a *Lactobacillus casei*-based microbiological assay, after enzymatic deconjugation by γ-glutamyl hydrolase [21].

### RNA isolation

Total RNA was extracted using the TRIZOL reagent (Invitrogen) starting with approximately 300 mg of frozen leaf material. RNA concentration and purity were quantified with Nanodrop measurements and the quality of the total RNA was checked on a 1% RNase free agarose gel.

Total RNA (5 ul) was treated with the DNase I Amplification Grade kit (Invitrogen) for digestion of single and double stranded DNA according to manufacturer's intructions.

Total RNA was cleaned using the RNeasy Mini Kit (Qiagen) starting with the 100 µl of DNase I treated RNA.The concentration of the cleaned RNA was measured and the samples were diluted with nuclease free water (Qiagen) to 400 ng/µl in a total volume of 10 µl.

### Microarray design

The distant pair design proposed for two colour microarrays experiments by 35 was followed and implemented in the R package designGG (http://gbic.biol.rug.nl/designGG/). This design uses genetic marker information to identify pairs of individuals with maximum dissimilarity across the mapping population and improves the efficiency of eQTL studies. In our study we used information obtained from 48 pairs of DH lines and the information on parental lines was additionally hybridized in two microarrays with dye swap of Cy3 and Cy5. The probes were classified in correspondence to the EST sequence origin: (1) *B.rapa*, (2) *B.napus*, (3) *B.oleracea* and (4) other *Brassica* species [27].

### QTL mapping analyses

QTL analysis was performed using the basic single marker regression procedure present in R/qtl [38]. This was done for both the expression ratio values and the metabolite datasets in a similar fashion, leading to results that could be easily combined in the end. A total of 78,688 expression probes together with the 228 reconstructed metabolites and the values of targeted metabolites (carotenoids,tocopherols and folic acid) were mapped back to the genetic map of *B. rapa* (Figures S1, S2, S3, S4) using the basic model. The expressions were measured using two-color array technology and for the mapping we used the ratio's between two genotypes 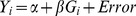
(Y_i_ =  Probe intensity, G_i_ =  Genetic effect).

In this model the genetic effect was annotated for the expression ratio's as described in [39]; β is the effect of the different allele (1 for A>B 0 for A =  = B and −1 A<B). This model was evaluated at each marker to get an estimate of the allelic effect on the expression probes. This results in a P-value, which was transformed into a LOD score. These LOD scores where then visualized in different ways to show underlying genetic architecture, by using QTL profile plots and heat maps.

Six single-copy genes with eQTLs detected using the microarray profiles were selected to compare results obtained from the microarray and real-time PCR (*BrARR3*_A09, *BrFRL2*_A09, and *BrCAM1*_A07, *BrCYCLIND1:1*_A02, *BrKRP2*_A03 and *BrDRL1*_A08). Transcripts of these genes were profiled with two technical replications using the RNA samples of the 92 DH68 lines that were previously used for microarray analysis, as described in [40], [37]. The eQTLs for these six single-copy genes identified using the microarray were confirmed by RT-qPCR, with higher LOD scores for RT-qPCR data.

We constructed the glucosinolates mQTL profile based on the metabolites detected by the untargeted LC-MS analysis. LOD scores for each marker were calculated using an MQM procedure [38]. The output of the transcriptional data of the candidate regulatory genes (eQTLs) was filtered for a LOD threshold value of >3.5. The data was analyzed using the MetaNetwork computational protocol as described in [41] to obtain second order correlation values. The generated files were plotted using Cytoscape [42].

### Identification of known regulatory genes for network analysis

To identify eQTL involved in the regulation of the biosynthesis of common *Brassica* phytochemicals, we first screened the probes represented on the microarray against a compiled list of genes that are known to be involved in the regulation of six biosynthetic pathways leading to the production of flavonoids, phenylpropanoids, glucosinolates, carotenoids, tocopherols and folate [28], [29], [43], [44], [45] (Table S1). The nucleotide sequences of the microarray probes were compared with the gene sequences of the current version of the *B. rapa* genome sequence available in the *Brassica* database (BRAD) (http://brassicadb.org/brad/) [46]. To identify cis and trans effects, the paralogues information of the selected candidate genes were also obtained from the BRAD website. The glucosinolate reference pathway was drawn in Cytoscape [42]. The reference KEGG pathway was imported after the implementation of the kgml reader plugin in Cytoscape.

## Results

### Genetic linkage map of DH68

A genetic linkage map was constructed for population DH 68. A total of 456 markers were mapped in the DH population (Figure S1). The total map length was 1233.221 cM and consisted of 10 linkage groups, corresponding to the 10 chromosomes of *Brassica rapa*. The largest linkage group was A03 with a size of 192.22 cM and the smallest linkage group was A04 with a size of 63.646 cM. Each of the linkage groups had at least one SSR marker, which allowed the identification of the corresponding chromosome and the comparison with previously published maps [31]. In addition to the SSR markers, gene targeted markers related to the glucosinolate pathway were mapped in this population as well. A total of 21 markers related to the glucosinolate (GLS) biosynthetic pathway were mapped in all the linkage groups except for A08, A05,A04 and A10. The linkage group with most GLS genes mapped was A03 with eight genes mapped. The mapping of this particular group of markers together with the SSRs was of aid for the identification of the map orientation and for further syntenic comparison with *Arabidopsis thaliana* in the search of candidate genes for metabolic pathways and cis/trans effects.

### Whole genome QTL analysis of metabolic content

Making use of targeted metabolic extraction and analyses procedures, including quantification using reference compounds, it was possible to measure the variation in absolute levels of selected health-related phytochemicals, i.e. tocopherols, carotenoids and folic acid, in the leaves of *B. rapa*. The parental line YS-143 produced significantly more of γ-tocopherol, α-tocopherol, lutein, β-carotene and folate than the parental line PC-175. Variation of carotenoids, tocopherols and folate within the DH population are presented in Table 1. A transgressive segregation for the quantity of content of these targeted compounds was observed: most of the levels were transgressive in a positive and negative direction. QTL analysis of these targeted compounds revealed significant mQTL regions LOD >3 distributed across the whole genome table S2. Overlapping QTL regions, with a possible regulatory function for more than one carotenoid, were located on linkage groups A03, A05 and A10. No significant results were found for δ-tocopherol. (Figures S2, S3, S4).

**Table 1:** Summary statistics of the metabolic variation for targeted metabolites (Tocopherols,Carotenoids and Folic acid).

	TOCOPHEROLS				CAROTENOIDS				
	βtoc	αtoc	γtoc	δtoc	βcarotene	Lutein	Neoxanthin	Violaxanthin	Folate
Mean	0.40	14.71	0.19	0.03	61.68	84.70	28.77	66.11	2304.53
Median	0.41	14.34	0.17	0.03	61.28	78.47	27.71	65.37	2183.27
Min	0.12	9.18	0.07	0.00	42.70	44.45	16.11	40.31	1303.68
Max	0.82	22.65	0.80	0.22	86.35	158.00	45.41	90.27	4115.69
*s.e.m	0.02	0.30	0.01	0.00	1.01	2.74	0.63	1.16	63.25
*%CV	36.04	18.92	58.23	104.29	15.41	30.36	20.50	16.44	26.04
PC	0.40	15.77	0.11	0.04	58.64	84.33	32.30	64.63	1434.00
YS	0.50	23.66	0.34	0.03	71.20	107.59	30.78	62.72	4208.00

Units for all the metabolites are in mg/kg per FW.*sem =  standard error of the mean, and %CV  =  coefficient of variation. Parental lines metabolites are shown PC =  pak choy, YS  =  yellow sarson.

The LC-MS untargeted profiling resulted in the identification of 228 reconstructed metabolites; from this set 41 were identified as corresponding to the flavonoids pathway (Table S3).

**Figure 1:**
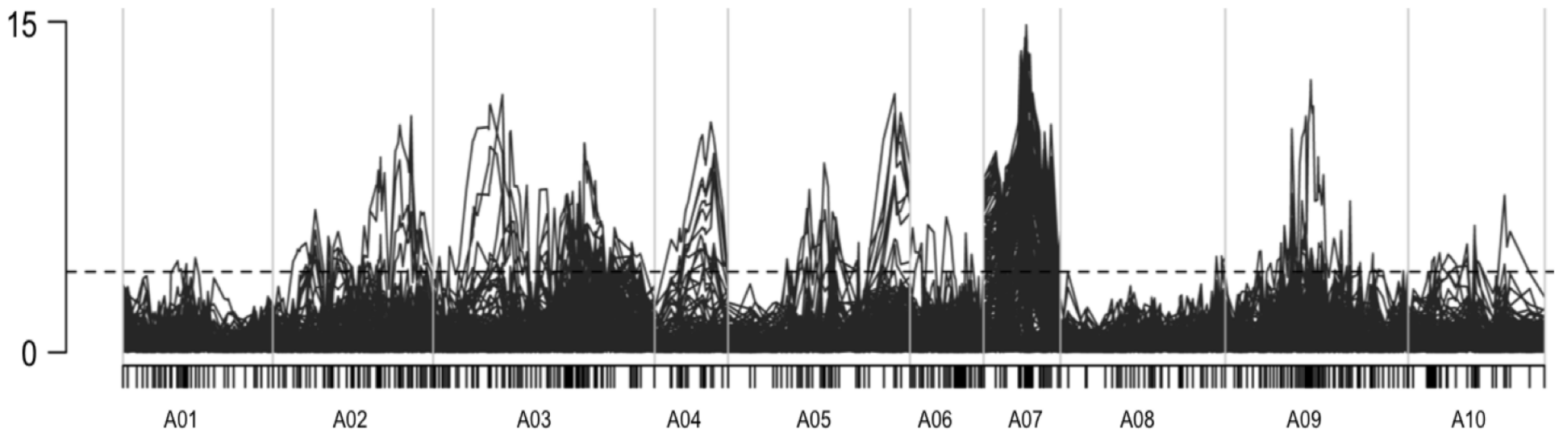
Whole genome QTL analysis of 228 reconstructed metabolites from the LC-MS data (mQTL). X-axis represent linkage group, and y-axis indicates lod score.

Significant mQTLs were detected for 166 out of the 228 reconstructed metabolites (i.e. 73%). These mQTL were not equally distributed over the *B. rapa* linkage groups, as coldspots and hotspots for the genetic regulation of metabolite content could be identified (Figure 1). The most important regulatory region was located on linkage group A07, where mQTLs were detected for 47% of all LC-MS metabolites.

### Whole genome QTL analysis of transcriptomics data and candidate gene filtering

**Figure 2:**
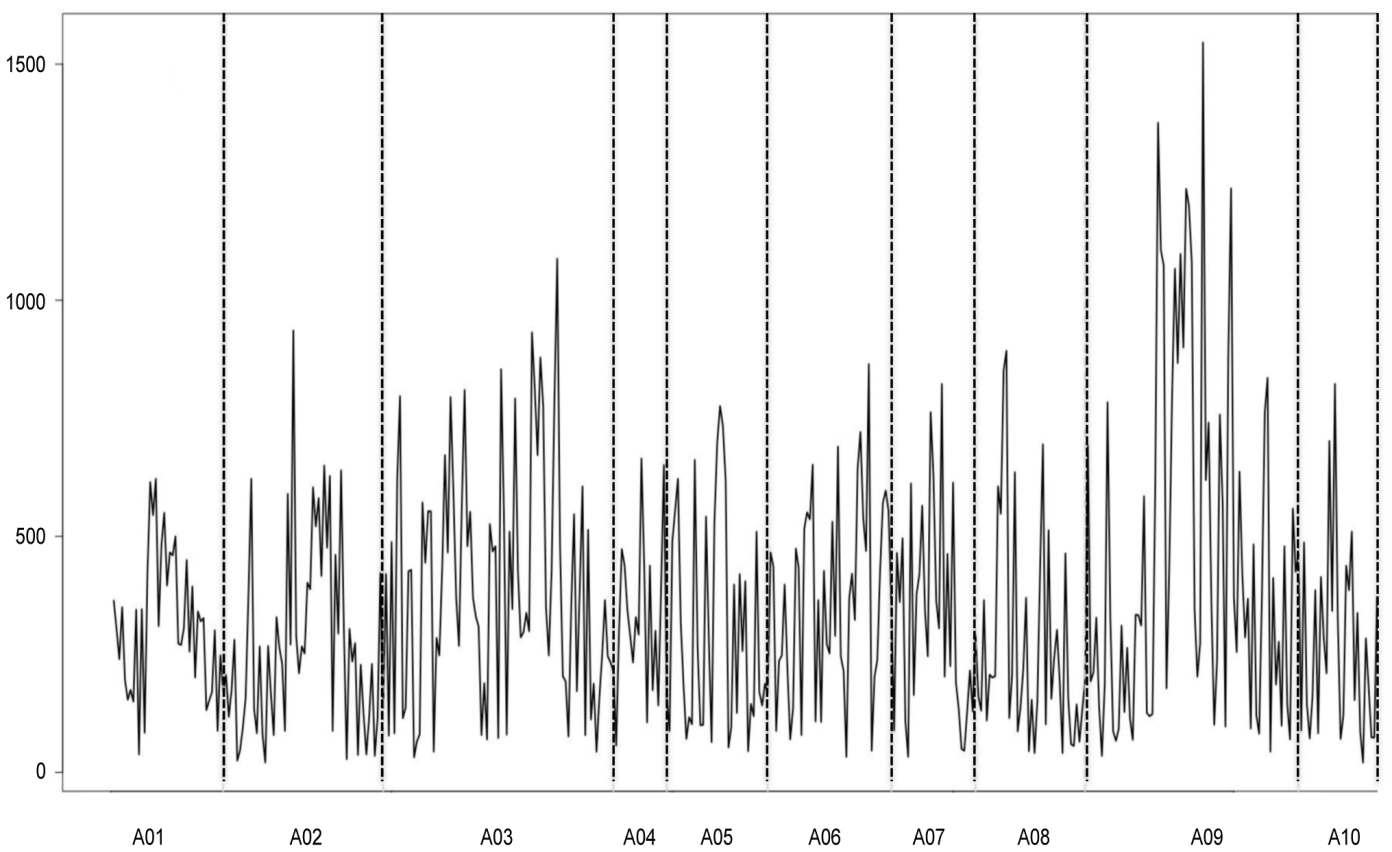
Whole genome QTL analysis of gene expression data (eQTL). On x-axis linkage group and on y-axis number of QTLs per map location.

We followed a regression analysis of the transcript abundance represented by 78,278 informative probes on the microarray against 456 mapped markers. In total, 44,358 probes were detected as significant against a genetic marker with a LOD score >3.5.The whole genome profile of the number of eQTL versus chromosome position indicates that there is no evidence of eQTL clustered as hotspots. Instead, the eQTL were distributed randomly across the genome with a higher number than average on linkage groups A02, A03 and A09. (Figure 2).

In general, to identify regulatory genes we searched for genomic regions in which metabolite levels (mQTL) seem to co-localize with significant LOD values of annotated probes (eQTL) corresponding to candidate genes of their biochemical pathway. While the region in linkage group A07 was detected as a hotspot for mQTLs, no eQTLs were observed among the known biosynthetic genes. However, when the complete set of probes with a significant LOD score of more than 3.5 in linkage group A07 was filtered for GO terms related to flavonoid regulation, we could identify three probes representing genes involved in positive regulation of flavonoid biosynthesis. (Table S4)

For most metabolites we could identify at least one eQTL overlapping with one mQTL. Only for α-tocopherol, having a QTL on linkage group A02, none of the annotated probes showed an eQTL at the same genomic region, and also for folic acid we were unable to identify probes that co-localize with its mQTL in A05 and A09, even when the selected LOD value of the annotated probes was lowered to LOD  = 3. In general we were able to identify cis and trans eQTLs for the target metabolites in several linkage groups. The eQTL results corresponding to each pathway are summarized in table S5 and figures S2, S3, S4.

**Figure 3:**
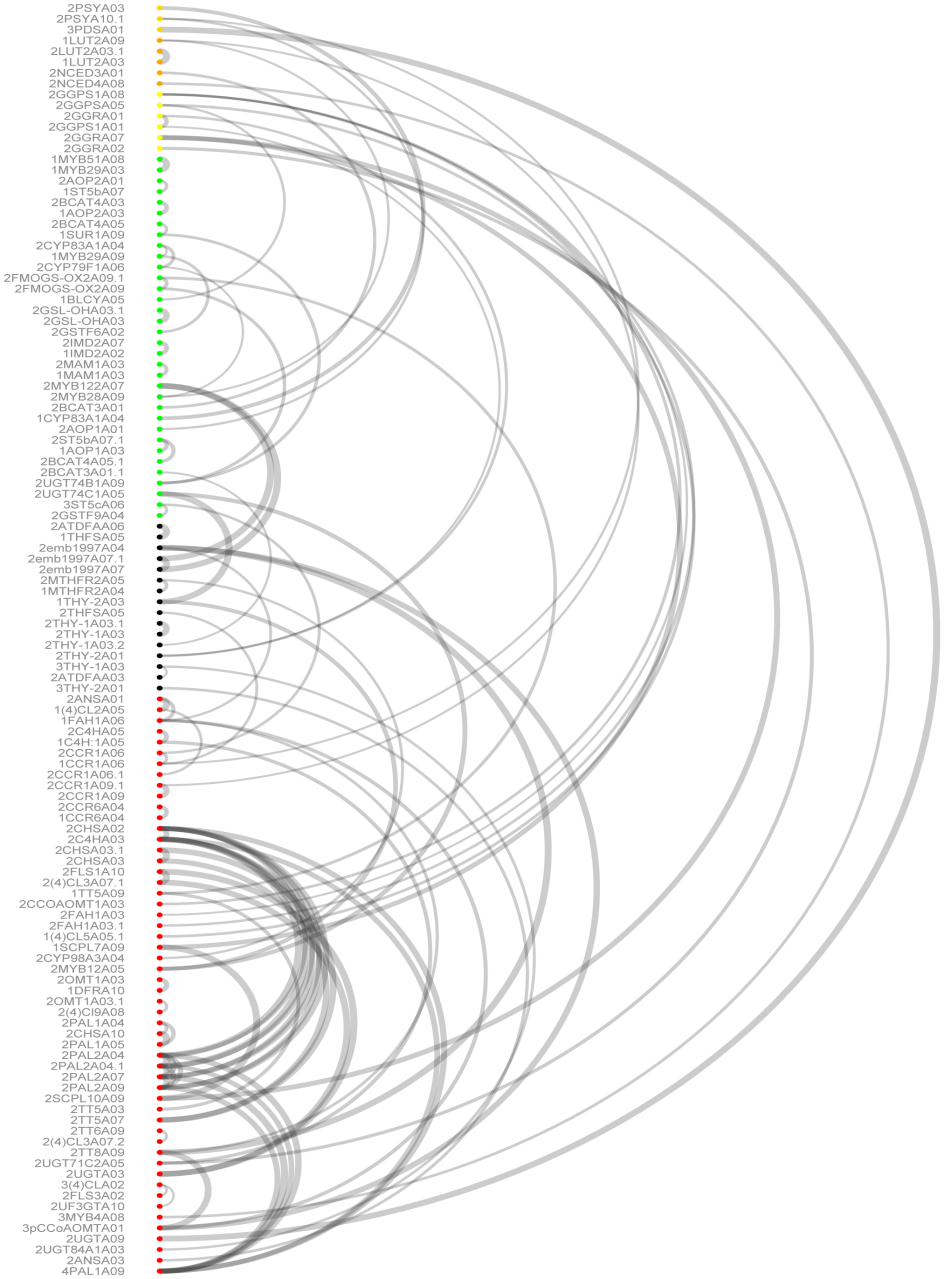
Correlation plot of eQTL location between metabolic pathways. Microarray probes representing pathway genes are represented by colored circles. Green: glucosinolates; red: phenylpropanoids/flavonoids orange: carotenoids; yellow: tocopherols; black: folate; dark yellow: tocopherols-carotenoids. Correlation values >0.3 are indicated.

Additionally, correlation analysis was performed between all the significant probes, annotated as known regulatory genes or transcription factors, with eQTL LOD scores >3.5 and a correlation value >0.3. The correlation analysis indicated a high within and between pathway correlation (Figure 3). The highest correlation values >0.8 were found between eQTLs results from different paralogs and between different genes belonging to the flavonoid pathway.

### Glucosinolate mQTLs Analysis

**Figure 4:**
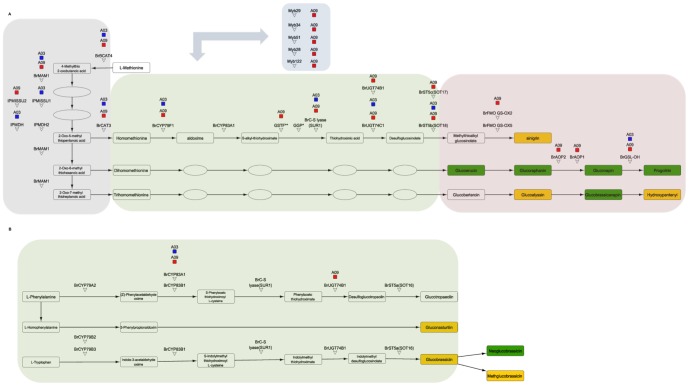
Reference Glucosinolate KEGG pathway. A. Cysteine and methionine metabolism. B. Phenylalanine, tyrosine and tryptophan biosynthesis.Yellow and green colored rectangles represent glucosinolates identified in the untargeted LC-MS dataset. Green colored rectangles indicate significant mQTL (lod values >3). Gray shading indicate side chain elongation step, green shading indicate core structure biosynthesis and red shading indicate the secondary modification steps.

To further analyze the regulatory network of a specific phytochemical pathway we focused on the very well characterized glucosinolate biosynthetic pathway (Figure 4).

The parental lines yellow sarson (R500) and a pak choi (PC175) are known to differ in their metabolic and genetic compositions [21], [22]. We detected 20 glucosinolates in the parental lines and within the DH population (Table S6). Significant mQTLs were detected for 13 out of the 20 glucosinolates, mostly representing aliphatic glucosinolates, while mQTLs were not detected for the aromatic glucosinolate and for two out of the three indolic glucosinolates.

**Figure 5:**
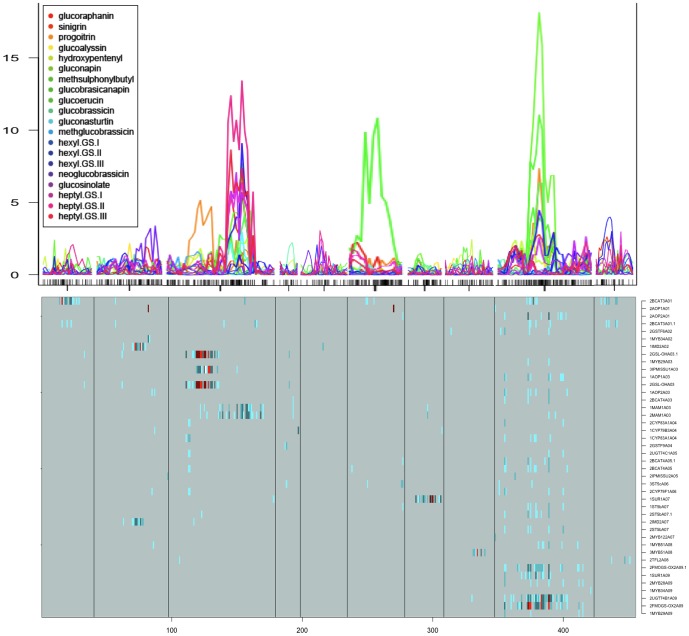
QTL analysis results of the glucosinolate pathway data. Top indicates mQTL and the bottom shows eQTL results of candidate genes. Names are presented on the right.Colours indicate different levels of significance: turquoise (logp  = 3), dark turquoise(logp  = 3–4), darkcyan (4–5), red (logp  = 5–7), dark red (logp  = 7–10), white (logp  = >10).

The mQTLs representing 12 aliphatic glucosinolates showed co-localization mostly in the genomic regions on linkage groups A03 and A09 (Figure 5). This co-localization indicates a high genetic correlation between glucosinolates. In the case of linkage group A03, mQTLs were found for the long chain aliphatic glucosinolates and in A09 the mQTLs were mostly detected for the short-chain (C3 to C5) aliphatic glucosinolates and their modified forms. In the case of the indolic glucosinolates we could identify one mQTL for neoglucobrassicin, which was located in linkage group A02. This mQTL had a lower LOD score value in comparison to the LOD scores for aliphatic glucosinolates.

### Transcriptional regulation of the glucosinolate pathway

**Figure 6:**
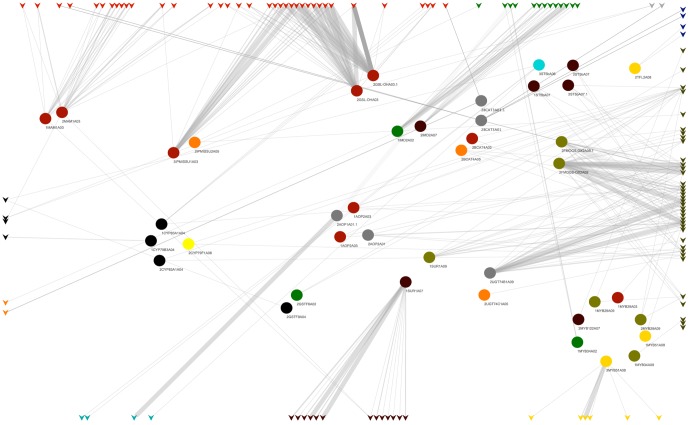
Metanetwork second order correlations results between eQTL and mapped markers. Colors indicate genomic location of the annotated microarray probe. Gray: A01, green: A02, red: A03, black: A04,orange: A05, turquoise: A06, brown: A07, yellow: A08, dark green: A09, blue: A10.

In an effort to enrich the genetic map with informative markers, we mapped genetic markers related to the glucosinolate pathway in the different linkage groups. Although we found significant gene expression QTLs (eQTLs) for the regulation of glucosinolate metabolism in all the chromosomes, two hot spots were identified in chromosome A03 and A09 (Figure 4, 6). Along linkage group A03 three positions were identified as important because of the co-localization of significant eQTLs. At top of chromosome A03 (38.4 cM) we mapped a genetic marker for the GLS transcription factor *Myb29*, while a putative eQTL representing *Myb 29* was found at 22.4 cM (with LOD of 2.1, which is lower than the significance threshold). At the Myb29 eQTL position at 22.4 cM, we found co-localizing eQTLs representing GLS biosynthesis genes: 2CYP79F1A06, 1-2CYP83A1A04, 2BCAT4A05, 2GSL-OHA03(2) and 2UGT74C1A05. We mapped a marker for GSL-OH at the middle of chromosome A03 (70.4cM), while the eQTL for the probe representing GSL-OH mapped in an interval spanning the same location. The GSL-OH eQTL interval co-located with the genetic map position of 3IPMISSU1A03 and 1MAM1A03. At the lower bottom position of A03, eQTL corresponding to 3IPMISSU1A03 and 1–2MAM1A03 colocalized at the position where a genetic marker for BCAT4 was mapped (121.1 cM). Finally, also in linkage A03, cis-regulation was found for the MAM gene, as the 1–2MAMA03 probe showed an eQTL at the position of the MAM genetic marker (126.0 cM). In addition, the same MAM probes had a significant eQTL at the position of a Myb28 marker (mapped at 127.2 cM on A03), although we did not find a Myb28-eQTL at this position.

Along linkage group A09 several regions were identified as eQTL hotspots. Many regions in linkage group A09 showed either colocalization of eQTLs or cis-effect of genes, which have been mapped at the corresponding positions in this population.

The Myb28 eQTL mapped at the Myb28 genetic locus (30.7 cM), where also significant eQTL colocalized for 1–2AOP2A01, 1AOP2A03, 2CYP79F1A06, 2CYP83A1A04, 1SUR1A09, 2BCAT4A03, 2BCAT4A05(2), 2FMOGS-OX2A09(2), 2GSTF6A02, 2UGT74B1A09, and 2ST5b(SOT18) A07 (2). The gene BrC-lyase (SUR1) was cis regulated, as eQTL (57.8 cM) and SUR1genetic marker (56.5 cM) colocalized. At the same position of the SUR1 eQTL the genes 2FMOGS-OX2A09 and 2UGT74B1A09 also had an eQTL. In an eQTL interval between 73.7 and 83.7cM on A09 the genes: 1AOP1A01, 2AOP2A01, 2GSTF6A02, 1AOP2A03, 2MAM1A03, 1MYB29A03, 2BCAT4A03, 2BCAT4A05(2), 2UGT74C1A05, 2CYP79F1A06, 1-2 (2) ST5b (SOT18) A07, 1MYB51A08, 2SUR2A08, 2MYB28A09, 1SUR1A09, 2FMOGS-OX2A09(2), 2BrBCAT3A01 and 2UGT74B1A09 had eQTL, suggesting that this is an important trans regulatory locus. Within this interval a genetic marker for UGT74B1 was also mapped, which could point to a regulatory role for this gene. Finally, within this linkage group at the position of the genetic marker for the transcription factor BrMYB51 (130.2 cM) significant eQTL were found for the following genes: 1MYB29A09, 2MYB28A09 and FMOGS-OX2A09 (2). The marker for FMOGS-OX5 was also mapped at the 136.8 cM position, and thus this gene is likely cis regulated.

### Glucosinolate Biosynthesis in *Brassica rapa*

The comparison of the mQTL and eQTL data revealed several GLS biosynthesis genes and transcription factors involved in the regulation of glucosinolate biosynthesis in *B. rapa* (Figure 6). In linkage group A03, for example, we were able to dissect the genetic function of the paralog of GSL-OH in A03 in the biosynthesis of progoitrin: its mQTL colocalized with both the eQTL and the genetic marker for 2GSL-OHA03 (2). In linkage group A03 at the large interval containing the eQTLs for 3IPMISSU1A03 and 1–2MAM1A03, the mQTLs for gluconapin, glucoraphanin, heptyl (I, II, III) and hexyl III glucosinolates co-localized within the region where genetic markers for the MAM gene and for the BrBCAT4 gene mapped. In linkage group A09 overlapping mQTLs were found for glucobrassicanapin, glucoerucin, gluconapin, hexyl-GLS III, methylsulphonylbutyl-GLS and progoitrin. These mQTLs co-localized at the interval containing several eQTLs and the genetic marker for UGT74B1.

In summary, for the eQTL analysis a total of 94 probes were selected as representatives of candidate genes for the GLS pathway. Forty-two from these 94 probes, representing 25 candidate genes, showed at least one eQTL. These genes had both cis and trans eQTL in most hotspots in the genomic regions at A03 and A09, with most of the genes showing trans-eQTL effects at the position of Myb29 in A03, and both UGT74B1 and Myb28 in A09.

## Discussion

A genetical genomics approach combining metabolic and expression data obtained from a DH population was chosen to gain insight into the genetic regulation of the metabolite composition in *B. rapa* leaves. To illustrate the strength of this approach, we selected six biosynthetic pathways involved in the production of health-related phytochemicals: carotenoids, tocopherols, folates, glucosinolates, flavonoids and phenylpropanoids. These pathways have very well been studied in *Arabidopsis thaliana* and most of the genes involved in these pathways have been characterized [28], [29], [43], [44] [47], [48], [49].

Traditionally, the synteny between *Brassica* and *Arabidopsis* and the recent availability of the *B. rapa* genome sequence have assisted in the prediction of candidate genes in genomic regions where a phenotypic QTL has been detected [25], [26], [31]. In the present study the use of large scale metabolomics approaches using both targeted and untargeted analytical platforms, combined with transcriptome analysis of a selection of annotated probes, allowed us to further analyze the QTL results and predict a group of candidate genes. The QTLs detected for many metabolites detected by untargeted LC-MS clustered within a genomic region at linkage group A7, indicating that these metabolites possibly shared a common genetic regulator.

To identify influential genes and gene products, the genetical genomics approach emerged as a tool to combine expression profiling with molecular marker analysis through the use of quantitative trait loci (QTL) analysis in a segregating population [14]. For our study we profiled the transcript abundance of the 92 DH lines with a microarray assembled from *B. napus*, *B. rapa* and *B. oleracea* EST sequences [27]. The direct comparison of metabolite mQTL and eQTL maps has shown the predictive capacity of eQTL to detect candidate genes for phenotypic differences in *Arabidopsis* and *B. rapa* [19]. This comparison between mQTL and eQTL in our study revealed co-localization of both in many cases. The predictive value of the QTL comparison through co-localization was very successful in the case of the isoprenoids and the glucosinolate pathway, but not in the case of both total folate and the phenylpropanoid and flavonoids with mQTL on A07. However, in the case of the flavonoids further inspection of significant eQTL (LOD>3.5) in linkage group A07 helped us to identify three genes with predicted function in flavonoids regulation in *Arabidopsis*.

To further analyze the regulatory network of a phytochemical pathway, we focused on the very well characterized pathway leading to the biosynthesis of glucosinolates. The variation and genetic regulation of the glucosinolate content has been widely studied [50], [51], [52], [53], [54], [55]) at different developmental stages and organs and with different approaches [49]. In the Ler vs Cvi RIL population of *Arabidopsis*, two major loci were found through QTL analyses to explain the observed variation for most of the aliphatic glucosinolates [11], [52], [53]. The MAM locus is responsible for the variation in chain length [56] and the AOP locus is responsible for the variation in side chain modification [53]. In a previous QTL study for glucosinolate variation in *B. rapa* leaves, a major QTL for the content of a number of aliphatic glucosinolates was identified on linkage group A03 in a DH population of a cross between a yellow sarson and a pak choi accession [31].

With the *B. rapa* sequence information available several candidate genes for glucosinolate regulation have been identified [28], [45]. However, these candidate genes have never been identified as loci underlying QTL and their functions have not been validated in *B. rapa*. In our study we were able to identify a metabolic network that clearly suggests a differential regulation of different glucosinolate classes. In the case of linkage group A03, mQTLs were detected for the long-chain aliphatic glucosinolates while at A09 mQTL were mostly detected for short-chain (C3 to C5) aliphatic glucosinolates and their modified forms. In these hotspot regions in A03 and A09 cis and trans effects were observed and most of the genes showed trans effects at the position of Myb29 in A03 and Myb28 - UGT74B1 in A09.

The analysis of expression differences, within a DH population, of a selected group of candidate genes for these selected pathways from the *Arabidopsis* research helped us to focus on well-known and characterized genes. Although these candidate genes are potential regulators of important pathways leading to metabolites of interest, the results indicated that the relevance of these genes is different in *B. rapa*. For example in our population the hotspot Myb28 - UGT74B1 in A09 is more relevant in *B. rapa* and also in *B. napus* seeds [57] than the AOP locus in *A. thaliana*. One difference that can have an effect in the QTL analysis is the triplication of the genomes in *Brassica* with the presence of paralogues. Our results with the presented genetical genomics approach indicate a possible sub-functionalization of the paralogues in *B. rapa*. For example, for the gene SUR1 we found two microarray probes annotated as corresponding to SUR1 copies mapping to linkage groups A07 and A09, with one copy showing significant eQTL values only at A07 (therefor called SUR1A07) and the other copy showing cis and trans eQTLS effects at A09 (thus called SUR1A09).

Significant genetic contribution to the regulation of the level of secondary metabolites in *B. rapa* opens the possibility for application of metabolic engineering in *Brassica* crops. However, it is important to realize that correlation between different pathways can exist, preventing the identification of a hotspot for regulation of specific metabolites. Our results of eQTL analysis indicated cross talk between several pathways, which means that they are interdependent, most likely as these pathways use common substrates (Figure 3). The data generated in our study is valuable for the selection of *B. rapa* accessions with higher or lower content of specific metabolites, which can help in marker-assisted selection for breeding purposes based on the identified candidate regulatory genes.

## Supporting Information

Figure S1 **Map Doubled Haploid DH68 population.**
(DOC)

Figure S2 **QTL analysis results of the carotenoids pathway data.** Top indicates QTL metabolic profiling and the bottom shows QTL expression results of microarray probes representing candidate genes, names are listed on the right. turquoise (logp  = 3), dark-turquoise (logp  = 3–4), dark-cyan (4–5), red (logp  = 5–7), dark-red (logp  = 7–10), white (logp  = >10).
(TIF)

Figure S3 **QTL analysis results of the tocopherols pathway data.** Top indicates QTL metabolic profiling and the bottom shows QTL expression results of microarray probes representing candidate genes, names are listed on the right. turquoise (logp  = 3), dark turquoise(logp  = 3–4), darkcyan (4–5), red (logp  = 5–7), darkred (logp  = 7–10), white(logp  = >10).
(TIF)

Figure S4 **QTL analysis results of the folates pathway data.** Top indicates QTL metabolic profiling and the bottom shows QTL expression results of microarray probes representing candidate genes, names are listed on the right. turquoise (logp  = 3), dark turquoise(logp  = 3–4), darkcyan (4–5), red (logp  = 5–7), darkred (logp  = 7–10), white(logp  = >10).
(TIF)

Table S1 **List of candidate genes for six biosynthetic pathways.** The probes of the microarray are identified with a genebank code, origin 1.*B.rapa*, 2.*B.napus*,3.*B.oleracea* and 4 other *Brassica* species. Abbreviation is included in whole genome eQTL profiling.
(XLSX)

Table S2 **Metabolite mQTL results are presented as LOD scores for each genetic marker after following an MQM procedure.** Centrotypes that were annotated are indicated at the top of the table.
(XLS)

Table S3 **List of annotated metabolites from the untargeted LCMS analyses, selected based on their significant QTL at A07.**
(XLS)

Table S4 **List of probes with significant LOD score values >3.5 in A07 with a GO-term related to flavonoid biosynthesis.**
(XLS)

Table S5 **Expression eQTL.** Results are indicated per metabolic pathway and according to the origin of the probe identified as candidate gene within each pathway. Paralogs of the significant eQTL were identified in *B.rapa*.
(XLSX)

Table S6 **List of glucosinolates identified in the population from a cross between pak choi PC-175 and yellow sarson YS-143.**
(XLSX)
