# Supplementary figures and images for: Regulatory Network of Secondary Metabolism in Brassica rapa: Insight into the Glucosinolate Pathway
Source: PLoS One. 2014 Sep 15;9(9):e107123. doi: 10.1371/journal.pone.0107123 (PMC4164526; doi:10.1371/journal.pone.0107123)

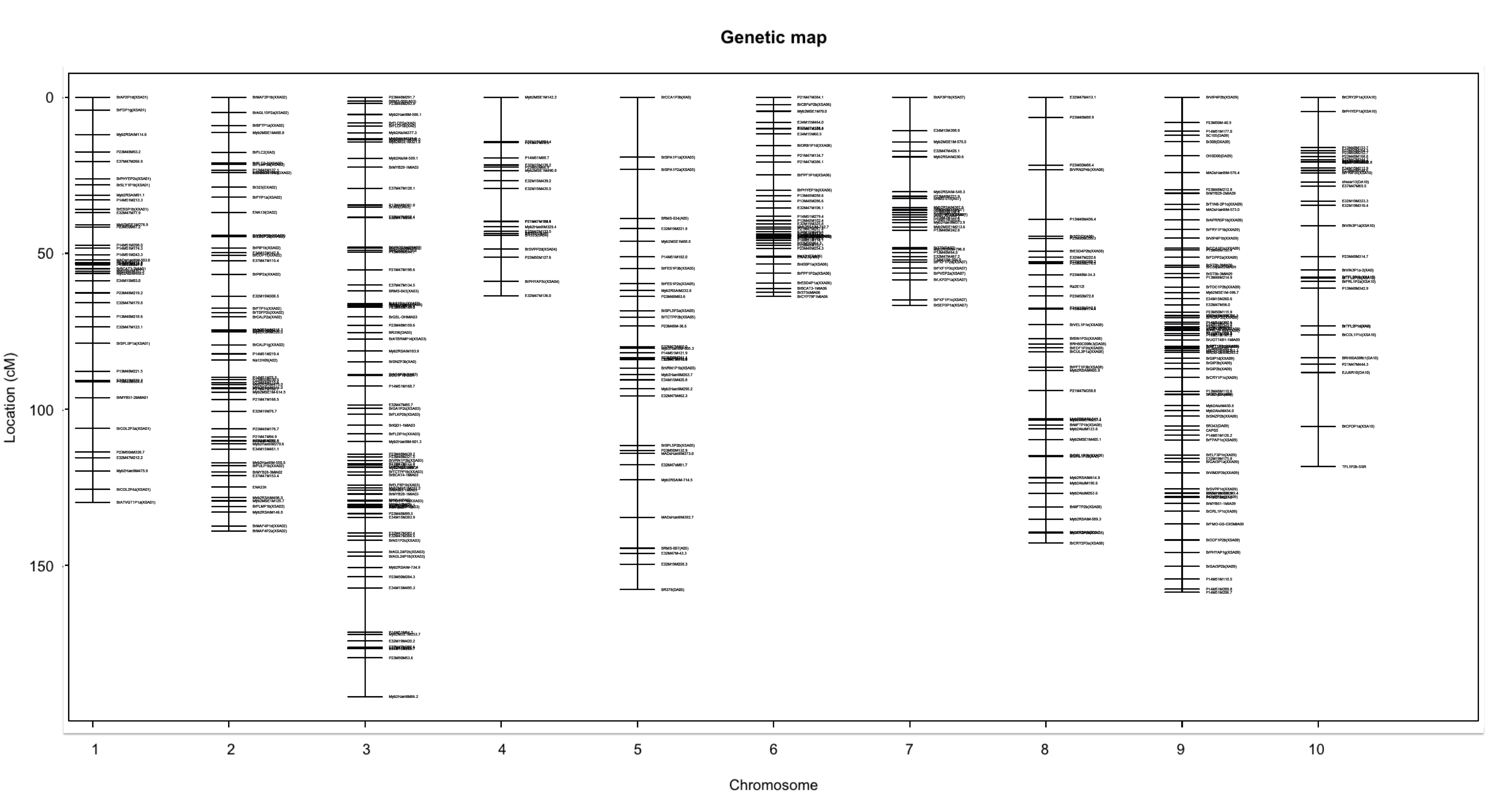


Genetic linkage map population DH 68

Supplement: Figure S1 — Map Doubled Haploid DH68 population. (DOC) [file pone.0107123.s001.doc]

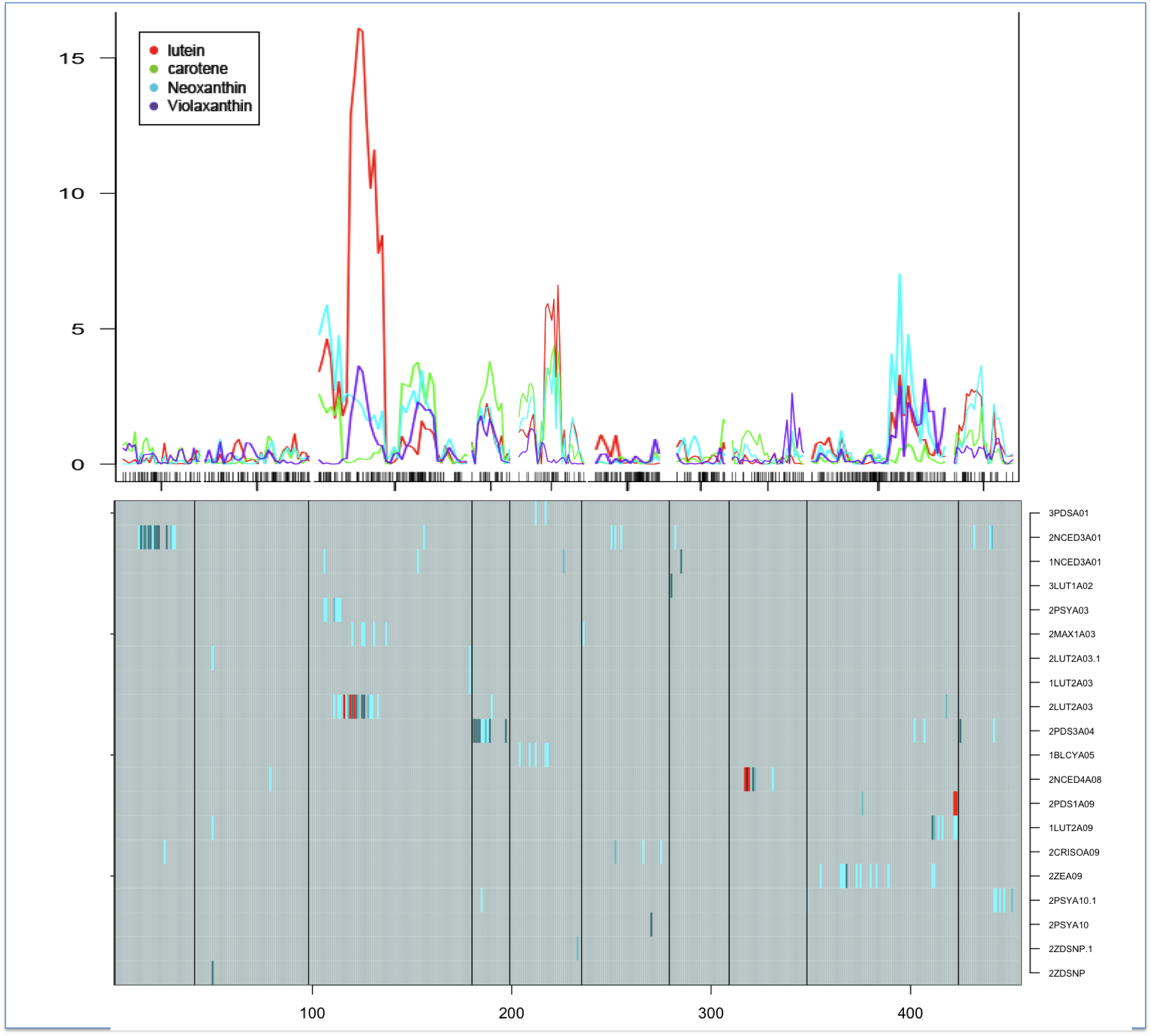

Supplement: Figure S2 — QTL analysis results of the carotenoids pathway data. Top indicates QTL metabolic profiling and the bottom shows QTL expression results of microarray probes representing candidate genes, names are listed on the right. turquoise (logp = 3), dark-turquoise (logp = 3–4), dark-cyan (4–5), red (logp = 5–7), dark-red (logp = 7–10), white (logp = >10). (TIF) [file pone.0107123.s002.tif]

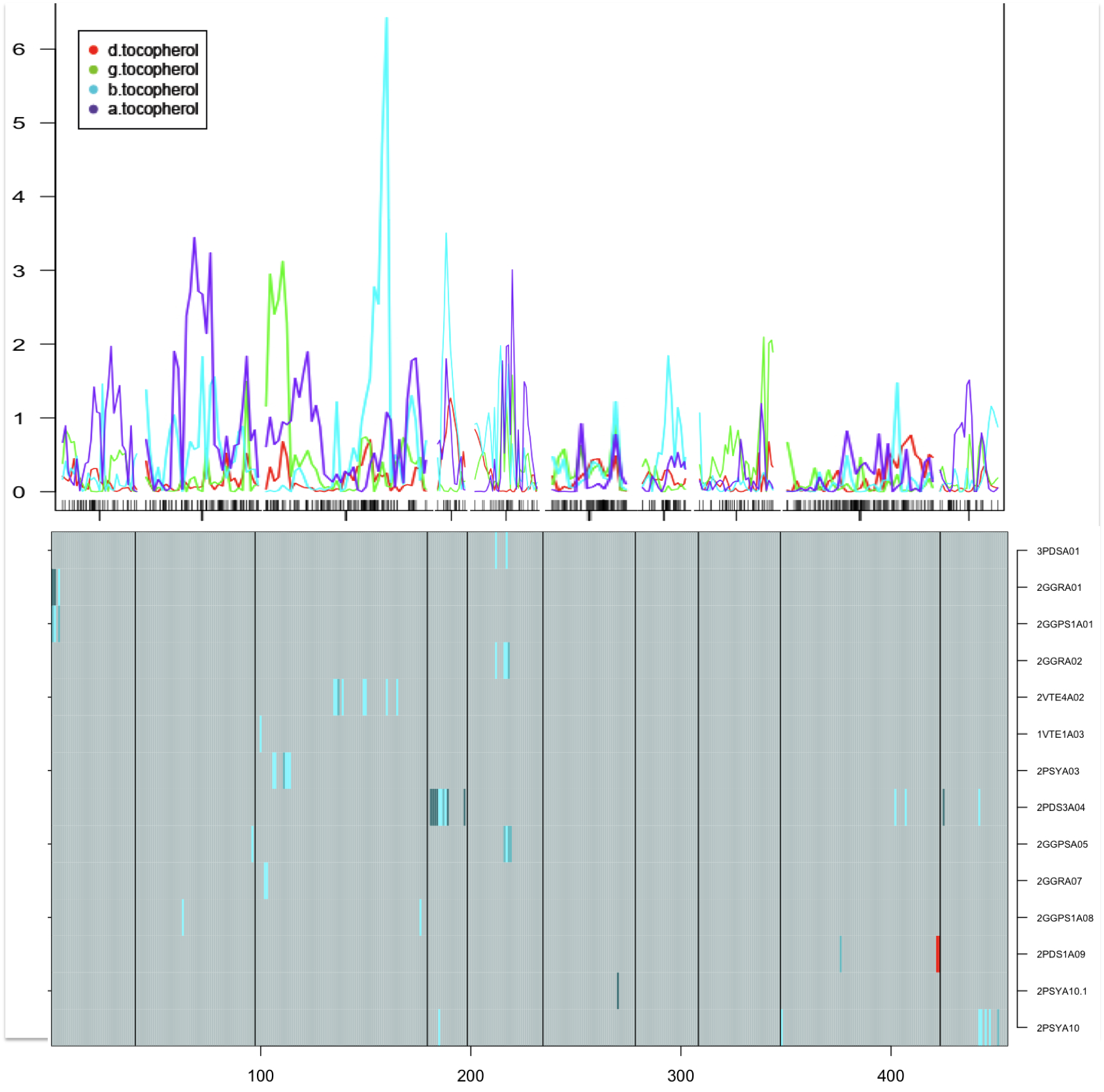

Supplement: Figure S3 — QTL analysis results of the tocopherols pathway data. Top indicates QTL metabolic profiling and the bottom shows QTL expression results of microarray probes representing candidate genes, names are listed on the right. turquoise (logp = 3), dark turquoise(logp = 3–4), darkcyan (4–5), red (logp = 5–7), darkred (logp = 7–10), white(logp = >10). (TIF) [file pone.0107123.s003.tif]

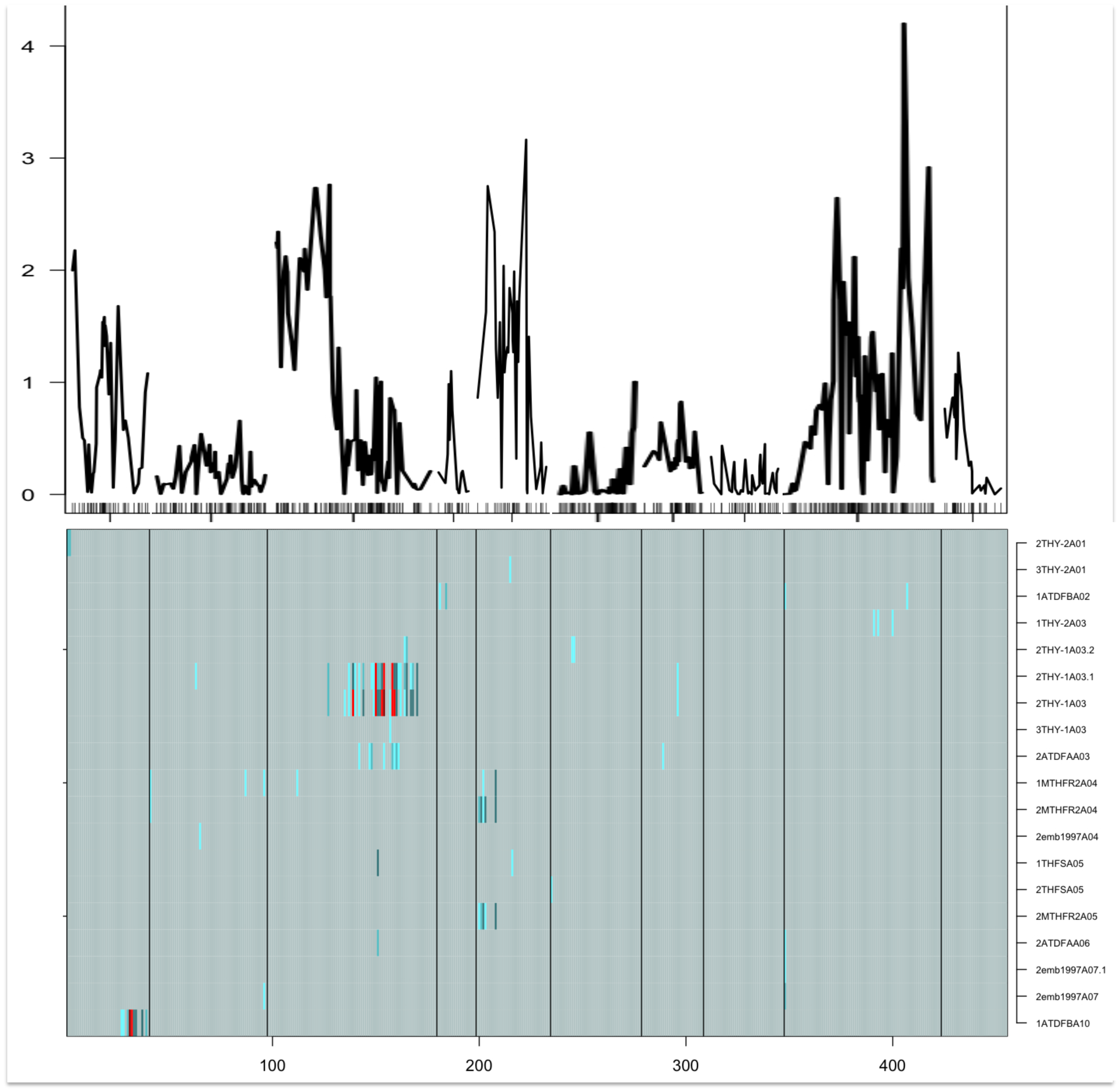

Supplement: Figure S4 — QTL analysis results of the folates pathway data. Top indicates QTL metabolic profiling and the bottom shows QTL expression results of microarray probes representing candidate genes, names are listed on the right. turquoise (logp = 3), dark turquoise(logp = 3–4), darkcyan (4–5), red (logp = 5–7), darkred (logp = 7–10), white(logp = >10). (TIF) [file pone.0107123.s004.tif]
